# Genetic diversity and population structure in the endangered tree *Hopea hainanensis* (Dipterocarpaceae) on Hainan Island, China

**DOI:** 10.1371/journal.pone.0241452

**Published:** 2020-11-30

**Authors:** Chen Wang, Xiang Ma, Mingxun Ren, Liang Tang

**Affiliations:** 1 Key Laboratory of Tropical Biological Resources of Ministry of Education, School of Life and Pharmaceutical Sciences, Hainan University, Haikou, China; 2 College of Ecology and Environment, Hainan University, Haikou, China; Chinese Academy of Forestry, CHINA

## Abstract

*Hopea hainanensis* Merrill & Chun (Dipterocarpaceae) is an endangered tree species restricted to Hainan Island, China and a small part of Northern Vietnam. On Hainan Island, it is an important indicator species for tropical forests. However, because of its highly valued timber, *H*. *hainanensis* has suffered from overexploitation, leading to a sharp population decline. To facilitate the conservation of this species, genetic diversity and population structure were assessed using 12 SSR markers for 10 populations sampled across Hainan Island. Compared to non-threatened *Hopea* species, *H*. *hainanensis* exhibited reduced overall genetic diversity and increased population differentiation (AMOVA: *F*_ST_ = 0.23). Bayesian model-based clustering and principal coordinate analysis consistently assigned *H*. *hainanensis* individuals into three genetic groups, which were found to be widespread and overlapping geographically. A Mantel test found no correlation between genetic and geographical distances (*r* = 0.040, *p* = 0.418). The observed genetic structure suggests that long-distance gene flow occurred among *H*. *hainanensis* populations prior to habitat fragmentation. A recent population bottleneck was revealed, which may cause rapid loss of genetic diversity and increased differentiation across populations. Based on these findings, appropriate strategies for the long-term conservation of the endangered species *H*. *hainanensis* are proposed.

## Introduction

Earth’s biodiversity is rapidly declining as a consequence of agricultural expansion, overexploitation, deforestation, pollution and climate change [1–3]. Approximately 40% of plant species are threatened with extinction [4]. Conservation genetics, a new discipline that applies the concepts and tools of population genetics to biological conservation, is aimed at preserving endangered species from extinction [1]. Endangered species are commonly characterized by small, fragmented populations and restricted gene flow among populations [3]. In small, isolated populations, mating occurs more frequently among relatives, and a shift to selfing may be observed in hermaphroditic plants. Inbreeding leads to homozygosity in detrimental recessive alleles and the consequent production of inferior offspring, a phenomenon known as inbreeding depression [5]. In addition, genetic drift is stronger in small populations, further contributing to the fixation of deleterious mutations and loss of genetic variation, which compromise the adaptive potential of a population and thereby increase its extinction risk [3, 6]. With the goal of studying the genetic diversity, population differentiation, mating system and historical demography of endangered species, the field of conservation genetics provides remarkable insights into the preservation of biodiversity in the real world [2].

Tree species from a single family, the Dipterocarpaceae, are key community members in Asian tropical forests, accounting for 20–50% of forest basal area and more than 50% of canopy trees [7, 8]. Many Dipterocarpaceae species represent important timber resources, and thus have been heavily exploited in tropical Asian countries. Due to massive logging for timber, as well as deforestation for agriculture, many dipterocarps are now classified as endangered or critically endangered [4, 8]. However, dipterocarp forests are far more than a mere resource for timber production. They are key components of Asian tropical rainforest ecosystems, serving as a foundation on which these highly diverse ecosystems are assembled. Indeed among 25 worldwide ‘biodiversity hotspots’, four are located in Southeast Asia [9]. Furthermore, dipterocarp forests provide a variety of ecosystem services and play a crucial role in maintaining the equilibrium of ecological processes at both regional and global scales [8, 10].

Dipterocarp forests flourish in the Malay Peninsula, Sumatra, Borneo, Java and wetter parts of the Philippines, and stretch to the northern limits of tropical Asia [8]. The species diversity of the Dipterocarpaceae is dramatically reduced at the range limits in contrast to the aseasonal equatorial zones of Malaysia and Indonesia [7, 11]. Hainan Island in China is located near the edge of the Asian tropics, and only two *Hopea* species, *H*. *hainanensis* Merrill & Chun and *H*. *reticulata* Tardieu, are found there. *H*. *hainanensis* grows in the tropical lowland forests of Hainan Island and Northern Vietnam. It is a large evergreen tree with a height up to 20 meters, and it blooms and fruits almost every year. This tree species serves as an indicator for tropical forests on Hainan Island [11, 12] and is known for its highly valued timber, which is extremely durable and suitable for building boats, bridges and houses. Polyploidy is infrequent in the family Dipterocarpaceae, but triploid and tetraploid species were recorded in genus *Hopea* [13, 14]. Although many dipterocarps have a mixed mating system, most mature seeds are outcrossed, indicating selective abortion of selfed fruits [8]. Neither ploidy nor mating system has been reported for *H*. *hainanensis*. The size of *H*. *hainanensis* populations on Hainan Island has been greatly reduced due to the overexploitation and habitat loss for rubber tree plantations and agriculture [15, 16]. The remaining populations are now severely fragmented, preserved only in a few natural reserves [11]. *H*. *hainanensis* was assessed as endangered in the IUCN Red List of Threatened Species [16] and classified as the first-class protective plant in the Information System of Chinese Rare and Endangered Plants (ISCREP) [17]. This species is quite scarce even in protected areas and the mature trees were estimated to be less than 250 [17]. However, the lack of information on the distribution of genetic variation in *H*. *hainanensis* has hampered the effective conservation and management of this endangered species.

In view of the ecological and silvicultural importance of the Dipterocarpaceae, there have been many studies of dipterocarp trees that have investigated patterns of genetic diversity, fine-scale spatial genetic structure, mating system and gene flow among populations [18–22] The majority of studied species have moderate to high levels of genetic variation, and low differentiation among populations, suggesting a historical pattern of outcrossing combined with large, stable populations [3, 8]. In addition, the genetic diversity and population structure of endangered species in the Dipterocarpaceae have been assessed for the purposes of conservation and management of genetic resources [23–26].

In this study, population genetic analyses of *H*. *hainanensis* were performed using 12 microsatellite markers newly developed for this species. The goal of the study was to quantify the amount of genetic variation in *H*. *hainanensis* populations on Hainan Island, and compare this to the genetic diversity of non-threatened *Hopea* species. Then, the geographic pattern of genetic variation was revealed and discussed. The effect of habitat fragmentation on genetic differentiation across populations was also evaluated. Our studies on genetic diversity and population structure should facilitate the long-term conservation of this endangered species.

## Materials and methods

### Sample collection

**Fig 1:**
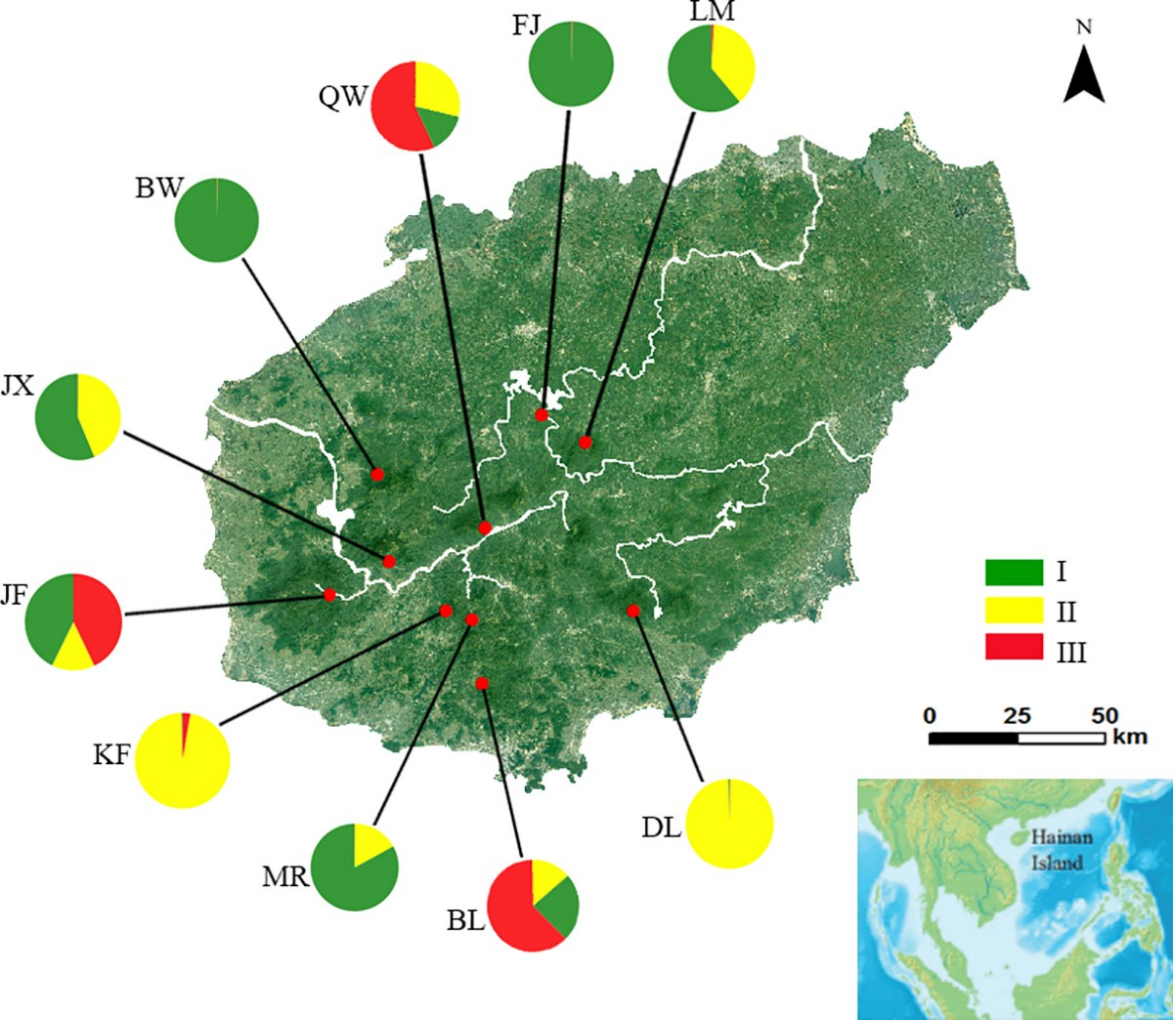
Geographic location of *Hopea hainanensis* populations used in this study (red dots). Pie charts illustrate the proportion of each of the three genetic clusters identified by STRUCTURE analyses for each population. Green, yellow and red represent genetic clusters I, II and III, respectively.

Totally 76 individuals were collected from 10 natural reserves for population genetic analyses, as *H*. *hainanensis* is very rare in tropical forests of Hainan Island [17] (Table 1, Fig 1). The field investigation was approved by the Administration Office of Nature Reserves, Forestry Department of Hainan Province. The habitat of *H*. *hainanensis* is primary or secondary tropical lowland forest, at altitudes ranging from 200 to 1000 meters [16]. Leaf samples were collected from mature trees with a diameter at breast height larger than 0.2 meter; all sampled trees were separated by a distance of at least ten meters. Young leaves free of disease and damage were collected and then immediately dried with silica gel. Voucher specimens for *H*. *hainanensis* were deposited in the Herbarium of Hainan University, Haikou, China.

**Table 1:** Sampling information for *Hopea hainanensis* populations on Hainan Island, China.

Population code	Sample size	Collection locality	Geographic coordinates	Voucher collection no.
BL	8	Baolong Forestry Station	18.4855°N, 109.4385°E	HBL10
BW	8	Bawang Mountain	19.0982°N, 109.1313°E	HBW09
DL	7	Diaoluo Mountain	18.6961°N, 109.8839°E	HDL06
FJ	9	Fanjia Country	19.2722°N, 109.6150°E	HFJ05
JF	7	Jianfeng Mountain	18.7422°N, 108.9902°E	HJF04
JX	9	Jiaxi Country	18.8429°N, 109.1662°E	HJX02
KF	7	Kafa Mountain	18.6988°N, 109.3303°E	HKF03
LM	8	Limu Mountain	19.1909°N, 109.7417°E	HLM01
MR	6	Maorui Forestry Station	18.6724°N, 109.4116°E	HMR08
QW	7	Qinwang Mountain	18.9388°N, 109.4468°E	HQW07

### DNA isolation, SSR amplification and genotyping

Total genomic DNA was extracted from the silica gel-dried leaves using a DNeasy Plant Mini Kit (QIAGEN, Shanghai, China) following the manufacturer’s instructions, with the addition of a step to remove high levels of polysaccharides in the initial extracts. The concentration and quality of the extracted DNA were measured with a NanoDrop 2000 spectrophotometer (NanoDrop Technologies, Inc., Wilmington, DE, United States). Using second generation sequencing and computational screening, twelve polymorphic SSR primer pairs were developed to genotype *H*. *hainanensis*. PCR amplification was carried out using an Eppendorf Mastercycler ep Gradient S thermocycler (Eppendorf, Hamburg, Germany); the 20 μL final reaction volume contained 2 μL gDNA (at least 50 μg/mL), 0.2 μL of each primer (50 μM) 10 μL 2× Taq PCR MasterMix (TIANGEN Biotech, Beijing, China), and ddH_2_O. The following cycling program was used: an initial five minute denaturation at 94°C; followed by 32 cycles of denaturing at 94°C for 20 s, annealing at 59–63°C for 20 s, and extension at 72°C for 60 s; with a final extension of seven minutes at 72°C. PCR products were mixed with Hi-Di Formamide loading buffer (Applied Biosystems, USA) and GeneScan 500 LIZ Size Standard (Life Technologies, Carlsbad, California, USA), and then genotyped on an ABI 3730XL DNA Analyzer (Applied Biosystems) owned by the TIANYI Biotechnology Company (Beijing, China). Alleles were scored using the GeneMarker software (SoftGenetics, State College, Pennsylvania, USA).

### Data analysis

In view of the autopolyploid nature of *H*. *hainanensis* (personal communication with Rong Wang of East China Normal University, who initiated the whole genome sequencing of *H*. *hainanensis*), allelic dosage was determined based on the ratios between peak intensities following the MAC-PR method [27] (S1 File). However, in the analysis of this autopolyploid, genotypic ambiguities caused by unknown allelic dosage could not be fully resolved with the MAC-PR method. In order to account for missing dosage information for partial heterozygotes, GenoDive version 3.04 [28] was used. Standard metrics of genetic diversity, such as the number of alleles (*N*_a_), effective number of alleles (*N*_e_), and observed (*H*_o_) and expected (*H*_e_) heterozygosity were then calculated. The heterozygosity-based *G*_is_ statistic was used to test for deviations from Hardy-Weinberg equilibrium (HWE). Another challenge posed by autopolyploidy is polysomic inheritance, under which double-reduction may occur, biasing the results of standard population genetic analyses [29]. A new software package, Polygene version 1.2b, was developed to analyze polyploid genetic data while taking into account both genotypic ambiguities and double-reduction [30]. Four polysomic inheritance models are implemented in the software, and the optimal model is selected based on the Bayesian information criterion (BIC). For each SSR locus, the possible genotypes and their posterior probabilities are inferred based on the allelic phenotype and inheritance model. The effects of null alleles, negative amplification and self-fertilization are also modeled by Polygene. To take advantage of these benefits, Polygene version 1.2b was implemented in this study to conduct further population genetic analyses.

The observed (*H*_o_) and expected (*H*_e_) heterozygosity, polymorphic information content (PIC) and inbreeding coefficient (*G*_is_) were estimated for each population and locus. Differentiation between populations was assessed using *G*_ST_ [31]. An analysis of molecular variance (AMOVA, [32]) was implemented in Polygene with modification for polysomic inheritance; AMOVA hierarchically partitions genetic variation among populations. Isolation by distance was assessed by regressing pairwise genetic distance against the natural logarithm of geographical distance (km) using the Mantel test [33] with 9,999 permutations. Slatkin's linearized *F*_ST_ was adopted as the measure of genetic distance [34]. Principal coordinate analysis (PCoA) was carried out based on Cavalli-Sforza’s (1967) chord distances [35], which have been shown to be the least biased distance measure when lacking dosage information [36].

A model-based clustering method, STRUCTURE v. 2.3.4 [37], was implemented to infer population structure, using an admixture model with correlated allele frequencies. Individuals were assigned to groups without prior knowledge of their population affinities [38]. The number of populations (*K*) varied from one to ten, and for each value of *K*, ten independent replicates were run with 100,000 burn-in iterations followed by 1,000,000 MCMC (Markov chain Monte Carlo) iterations. The best *K* was inferred using STRUCTURE Harvester [39] following recommended procedures [40]. The Greedy algorithm of Clumpp version 1.1.2 [41] was used to permute the independent repetitions for the selected value of *K*, and a graphical representation of the population structure was generated with Distruct version 1.1 [42]. A neighbor-joining tree based on Cavalli-Sforza’s (1967) genetic distance was created for the *H*. *hainanensis* individuals using the software package MEGA 5.0 [43].

A qualitative graphical method was performed to identify potential population bottleneck in *H*. *hainanensis* [44]. Allele frequencies were estimated using Polygene under the best polysomic inheritance model. SSR alleles were grouped into each of 10 allele frequency classes and then a frequency histogram was plotted. The 10 allele frequency classes are 0.001–0.100, 0.101–0.200, 0.201–0.300, etc. The rationale of the graphical method is that compared to common alleles, rare alleles are expected to be lost rapidly during a bottleneck. As a result, alleles at low frequency (<0.1) would be less abundant than alleles at intermediate frequency (e.g. 0.101–0.200) after population bottleneck, regardless of the mutation rate and model [44]. For comparison, SSR data of three dipterocarp species, a critically endangered tree of the Seychelles, *Vateriopsis seychellarum* [23], and two non-threatened species in the rainforests of Southeast Asia, *Shorea leprosula* [45] and *S*. *macrophylla* [21], were further analyzed with the qualitative graphical method.

## Results

Based on the BIC, the optimal polysomic inheritance model selected for Polygene analyses is RCS (S1 Table). For the 12 SSR markers genotyped across the ten *Hopea hainanensis* populations on Hainan Island, a total of 45 alleles were detected, for an average of 3.75 alleles per locus; the minimum number of alleles detected per locus was two (at loci Hha1, Hha9 and Hha10) and the maximum was six (Hha7 and Hha8). The polymorphism information content (PIC) varied from 0.205 (Hha10) to 0.707 (Hha7), with an average of 0.477. A large number of loci were found to deviate from HWE (*P* < 0.05) across the study populations (Table 2). At the population-level, the effective number of alleles (*N*_e_) ranged from 1.550 in DL to 2.351 in JF, averaging 1.964 alleles per population. The expected heterozygosity (*H*_e_) as estimated by GenoDive ranged from 0.265 (DL) to 0.525 (JF), while the *H*_e_ estimated by Polygene ranged from 0.233 (DL) to 0.495 (JF). The inbreeding coefficients (*G*_is_) were all greater than zero, ranging from 0.138 (FJ) to 0.408 (JF), and averaging 0.243 (Table 3).

**Table 2:** Summary statistics for the 12 SSR markers across all *Hopea hainanensis* populations with metrics of genetic diversity estimated by GenoDive 3.04 and Polygene version 1.2b.

Locus	Sequences (5'–3')	Repeat motif	Allele size	GENODIVE					POLYGENE			
				*N*a	*N*e	*H*o	*H*e	dHWE	*H*o	*H*e	PIC	*G*_is_
Hha1	F: AGTTGGAGATTAAAGAAAGTGGCT	(TTTTA)_6_	103–108	2	1.476	0.423	0.335	7	0.428	0.336	0.280	-0.272
	R: TTCAATTTAGACCCGTGGACCTC											
Hha2	F: ACATGGTCTTTGTTATCTGCTTA	(TTCT)_7_	155–163	3	1.899	0.552	0.579	5	0.566	0.573	0.507	0.012
	R: CCATGGTGCTACAACCTTTCTTG											
Hha3	F: TTCATGGTCATTGAGTCATAGGT	(AT)_10_	124–134	4	2.010	0.392	0.629	6	0.393	0.579	0.532	0.321
	R: GCCTCTACCTAGTGTATGAAGGC											
Hha4	F: ACCTGGTAAGCCATAACACTGAA	(TTC)_6_	144–150	3	2.712	0.755	0.661	9	0.756	0.655	0.581	-0.155
	R: TGATGCAAGCTCCAGAAACAAAG											
Hha5	F: AGTCAATGAGAAGGAGACATGTT	(TA)_8_	116–132	3	1.318	0.000	0.422	5	0.000	0.381	0.339	1.000
	R: AAGTCATTTGGTAAAAGGTGCCC											
Hha6	F: GCTTTCTGCATTTCCTTGAGAGA	(AT)_9_	141–153	4	1.200	0.060	0.504	4	0.064	0.455	0.416	0.858
	R: TGATTAGCTGCTGAATTTGGCTG											
Hha7	F: ACGAATGGAGGTTTGTAATTGGA	(AT)_10_	127–137	6	2.144	0.405	0.772	8	0.411	0.747	0.707	0.449
	R: AGAGTACAATCGGGATCAATGGA											
Hha8	F: TCAAACGCAACATGGAATAAGGA	(AT)_9_	222–230	6	2.555	0.503	0.751	8	0.505	0.735	0.691	0.313
	R: AGCCATTAACTCAGAACACGAGA											
Hha9	F: GATGAGGGATAATGGTGCGTTTG	(AAG)_5_	126–129	2	1.383	0.111	0.468	5	0.104	0.448	0.348	0.767
	R: CAACTCACGCCTCTGTGTTATTG											
Hha10	F: TCAATCGTTTTGAACCACAGGTG	(AT)_8_	157–159	2	1.244	0.211	0.237	1	0.202	0.232	0.205	0.129
	R: AGCTATTGCCTAGAAGATTTCACAC											
Hha11	F: GGCATCGTAATACCGCATAGAGA	(AT)_10_	157–165	5	1.965	0.455	0.680	4	0.458	0.657	0.607	0.303
	R: CTACCAACAACACTAGGCGCTGT											
Hha12	F: ATTACTAACCTTTGCCCACTCCT	(GT)_10_	78–94	5	1.865	0.530	0.565	4	0.530	0.568	0.510	0.066
	R: ACCAGCTTTAGCCAATTCAAACC											

*N*_a_: number of alleles for each marker across all populations, *N*_e_: effective number of alleles for each marker across all populations, *H*_o_: observed heterozygosity, *H*_e_: expected heterozygosity, dHWE: number of populations deviating from Hardy–Weinberg equilibrium (*P* < 0.05), PIC: polymorphic information content, *G*_is_: inbreeding coefficient.

**Table 3:** Genetic diversity of *Hopea hainanensis* populations based on 12 SSR markers.

Population	*N*	GenoDive					Polygene			
		*N*a	*N*e	*H*o	*H*e	dHWE	*H*o	*H*e	PIC	*G*_is_
BL	8	2.917	2.045	0.357	0.495	7	0.356	0.477	0.414	0.258
BW	8	1.833	1.780	0.444	0.355	6	0.444	0.345	0.278	0.139
DL	7	1.583	1.550	0.294	0.265	6	0.292	0.233	0.188	0.367
FJ	9	1.833	1.792	0.441	0.357	7	0.440	0.341	0.275	0.138
JF	7	2.917	2.351	0.295	0.525	8	0.295	0.495	0.433	0.408
JX	9	2.750	2.109	0.373	0.459	8	0.372	0.445	0.387	0.205
KF	7	2.667	1.900	0.348	0.419	5	0.347	0.402	0.345	0.197
LM	8	2.667	2.100	0.425	0.487	6	0.423	0.487	0.418	0.153
MR	6	2.500	1.970	0.385	0.419	7	0.384	0.399	0.347	0.223
QW	7	2.917	2.041	0.304	0.491	6	0.302	0.469	0.405	0.340
Average (SD)	7.2	2.458 (0.511)	1.964 (0.221)	0.367 (0.058)	0.427 (0.081)		0.366 (0.058)	0.409 (0.084)	0.349 (0.080)	0.243 (0.098)

*N*_a_: number of alleles in a population, *N*_e_: effective number of alleles in a population, *H*_o_: observed heterozygosity, *H*_e_: expected heterozygosity, dHWE: number of populations deviating from Hardy–Weinberg equilibrium (*P* < 0.05), PIC: polymorphic information content, *G*_is_: inbreeding coefficient.

The overall *G*_ST_ across all populations and loci was 0.229. Pairwise comparisons of genetic differentiation between populations showed that *G*_ST_ ranged from 0.001, between populations BW and FJ, up to 0.271, between populations BW and DL. Some populations, for example QW, KF and DL, were highly divergent from the other populations (S2 Table). The population genetic structure of *H*. *hainanensis* on Hainan Island did not conform to the isolation by distance model. No correlation was detected (Mantel test: *r* = 0.040, *p* = 0.418) between the genetic distance (as measured by Slatkin's linearized *F*_ST_) and the natural logarithm-transformed geographical distance among populations (S1 Fig). An analysis of molecular variance (AMOVA) revealed that 77.43% of total genetic variation was found within populations, while a small portion of variation (22.57%) was attributed to among population differentiation (Table 4).

**Table 4:** Analysis of molecular variance (AMOVA) for *Hopea hainanensis* populations.

Source	df	Sum of squares	Variance	Percentage of variation	*F*-statistic
Among populations	108	7980.81	2.06	22.57	*F*_ST_ = 0.23
Among individuals within population	764	10111.39	2.05	22.47	*F*_IS_ = 0.29
Within individuals	2652	13319.49	5.02	54.96	*F*_IT_ = 0.45

All variance components were statistically significant (P < 0.005); df: degrees of freedom.

**Fig 2:**
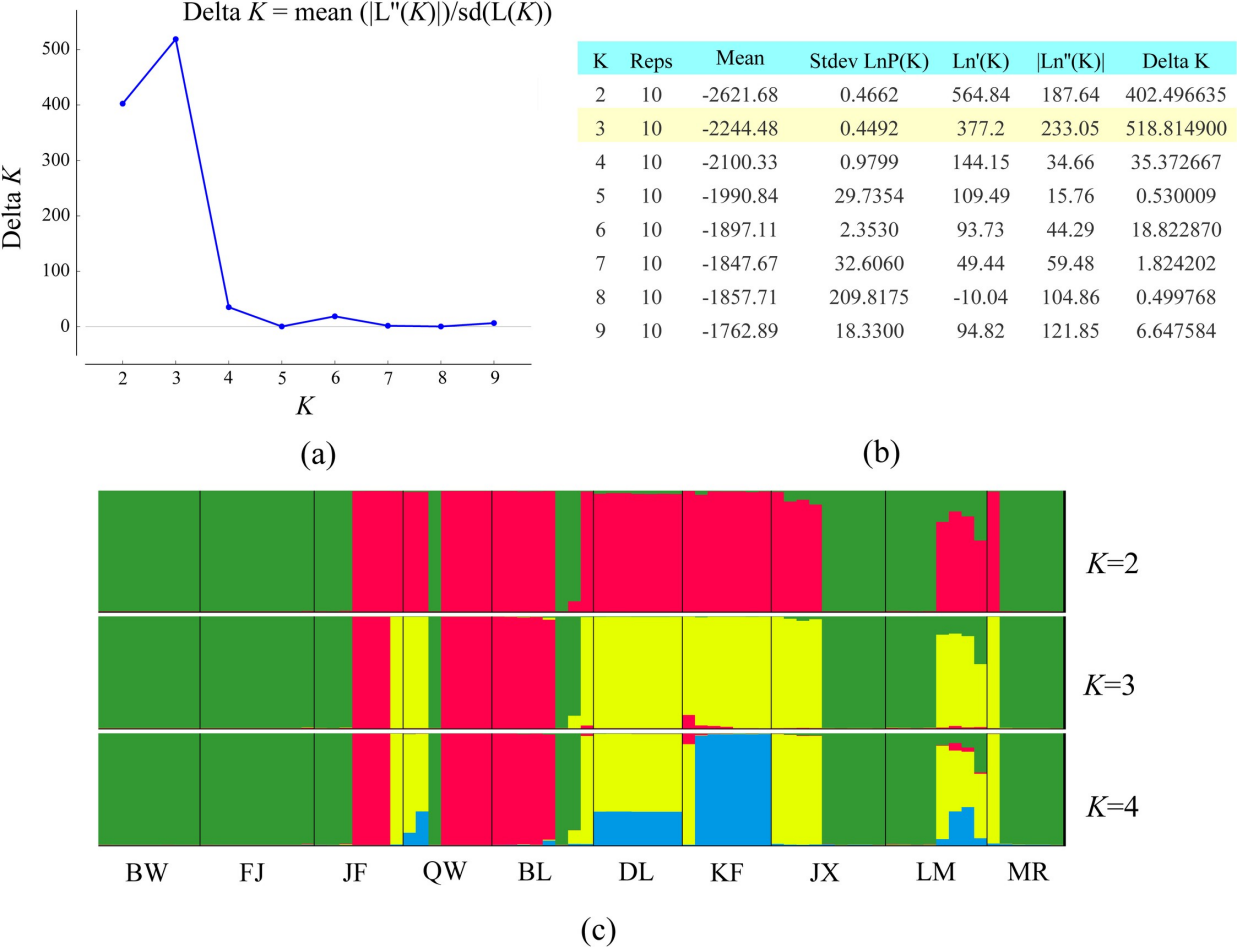
Results of the STRUCTURE analysis. (a) Using the Δ*K* method, which is based on the rate of change in the log probability of the data between successive *K* values, the most likely number of clusters was *K* = 3. (b) Log probabilities and Δ*K* values for *K* from two to nine. (c) The results of individual assignment at *K* = 2, 3 and 4. Each vertical bar represents an individual, and the proportion of the colors corresponds to the posterior probability of assignment to one of the three genetic clusters.

In model-based STRUCTURE analysis, LnP(*K*) was found to increase from *K* = 1 to 10 and Δ*K* was maximized at *K* = 3, suggesting that there are three distinct genetic clusters in *H*. *hainanensis*. The results of the individual assignment indicated that only a few individuals were found to have mixed ancestry of more than one genetic cluster (Fig 2). Five populations, FJ, BW, DL, KF and MR, were dominated by one genetic subpopulation. In LM and JX, two genetic subpopulations were represented in roughly equal proportions. Lastly, in JF, QW and BL, all three genetic subpopulations were represented, with one cluster (red, III) dominated (Fig 1). Geographically, individuals assigned to the same genetic cluster were widespread, and the distributions of the three genetic clusters were overlapping (Fig 1).

**Fig 3:**
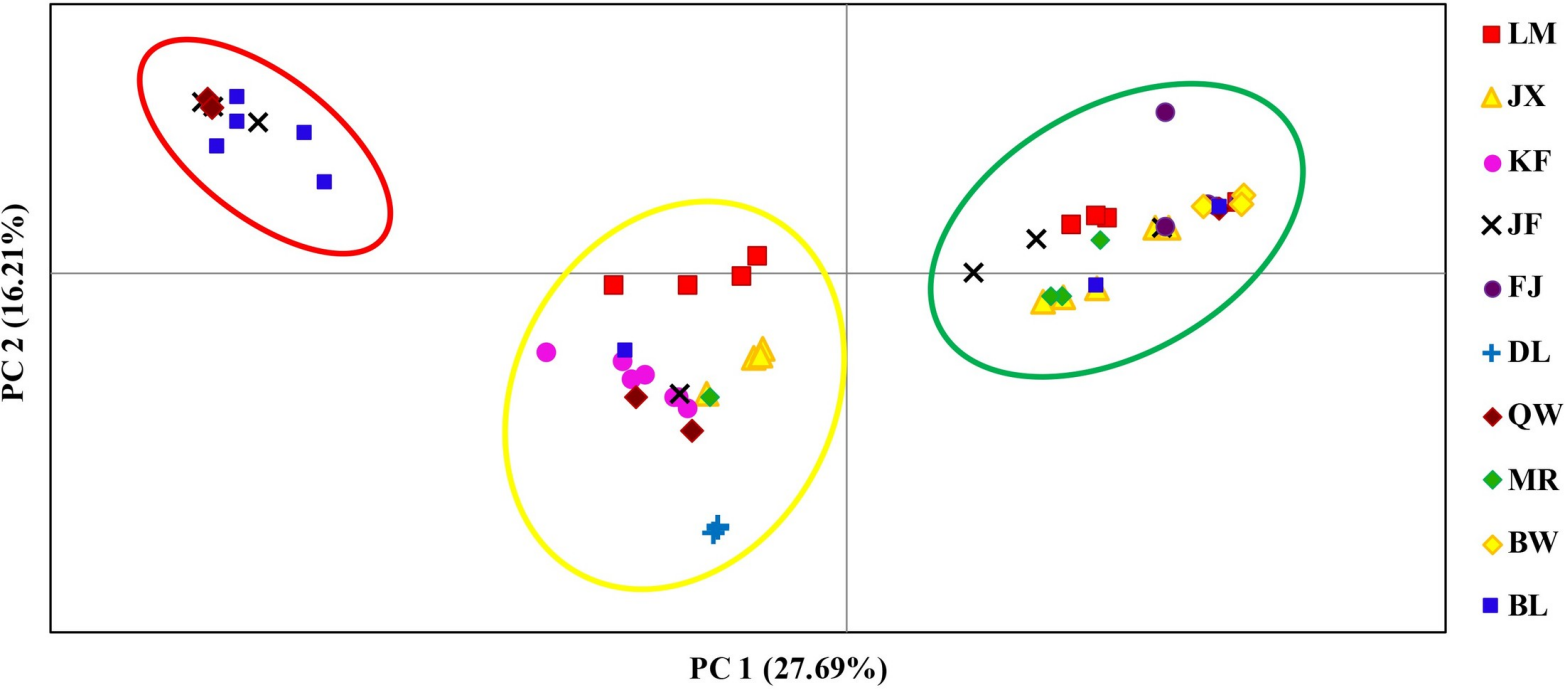
Principal coordinate analysis (PCoA) based on Cavalli-Sforza’s (1967) chord distance among individual samples of *Hopea hainanensis*. The three genetic groups identified by PCoA (highlighted by green, yellow and red ellipses) were consistent with the STRUCTURE results when *K* = 3.

The genetic relationships among *H*. *hainanensis* individuals were further explored using PCoA, and the results were consistent with the STRUCTURE analysis (Fig 3). The first two principal coordinates explained 43.89% of the total genetic variance (27.69% and 16.21%, respectively). Three genetic groups with clear boundaries were identified, corresponding to the three genetic components determined in the STRUCTURE analysis. In addition, using Cavalli-Sforza’s (1967) chord distances, a neighbor-joining tree was constructed. In the tree, individuals were divided into three groups, again in agreement with the three genetic groups identified by both PCoA and STRUCTURE (S2 Fig).

**Fig 4:**
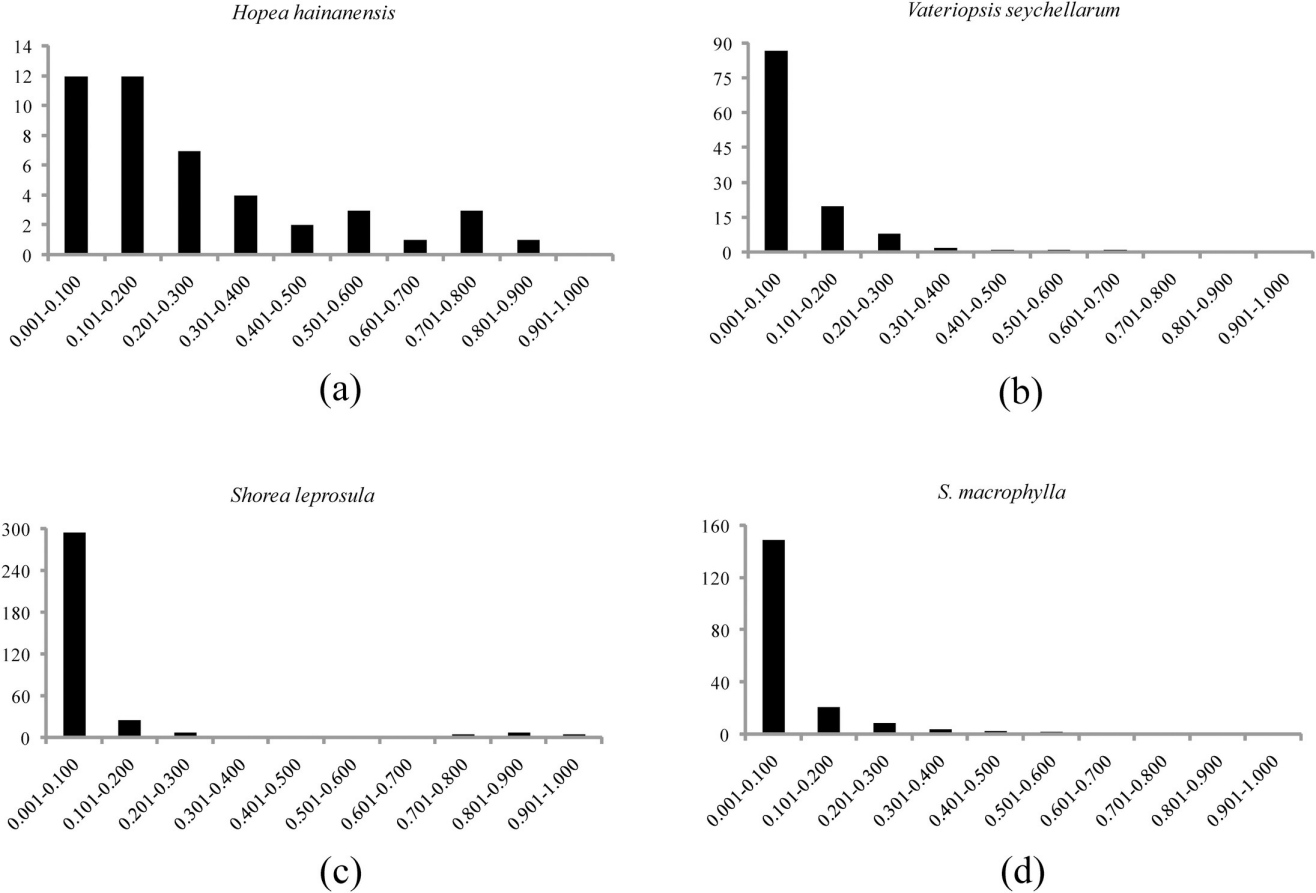
The allele frequency distributions based on SSR data from *Hopea hainanensis*, *Vateriopsis seychellarum*, *Shorea leprosula* and *S*. *macrophylla*.

The distribution of allele frequencies in *H*. *hainanensis* is obvious distorted and different from the other three dipterocarp species (Fig 4). The number of alleles at low frequency (<0.1) was equal to the number of alleles at intermediate frequency (0.101–0.200) in *H*. *hainanensis*. However, the alleles at low frequency (<0.1) are much more abundant than those at intermediate frequency (0.101–0.200) in the other two non-threatened *Shorea* species. Such a mode-shift distortion of allele frequency distribution is a characteristic of bottlenecked populations, revealing that *H*. *hainanensis* on Hainan Island may have undergone a recent population bottleneck.

## Discussion

### Genetic diversity in *Hopea hainanensis*

Species in the family Dipterocarpaceae are generally characterized by moderate to high levels of genetic diversity and low differentiation among populations [8]. This pattern of genetic variation is expected for long-lived, perennial woody plants with predominantly outcrossing mating systems [46]. For example, using SSR markers, most *Shorea* species have been found to have high levels of genetic variation (as measured by expected heterozygosity [*H*_e_] and the number of alleles per locus) [19, 47–49]. *Hopea dryobalanoides* is a non-threatened species widely distributed in Malaysia, Sumatra and Borneo [50]. A single population of *H*. *dryobalanoides* was collected from the Pasoh Forest Reserve in Peninsular Malaysia, and five SSR markers were used to evaluate its genetic diversity. Expected heterozygosity (*H*_e_) was estimated at 0.678 in *H*. *dryobalanoides*, which is comparable with that found for a number of *Shorea* species [21, 22, 49]. In contrast, in this study, the average *H*_e_ for *H*. *hainanensis* populations from Hainan Island was 0.409 as estimated by Polygene and 0.427 as estimated by GenoDive, which is markedly lower than the *H*_e_ measured for *H*. *dryobalanoides* [50]. Furthermore, fewer alleles were discovered in *H*. *hainanensis* (*N*_a_ = 2.46) versus *H*. *dryobalanoides* (*N*_a_ = 5.60). In most cases, reductions in the effective population size and shifts of mating system from outcrossing to selfing cause the loss of genetic diversity within species [51, 52]. The low genetic diversity within populations of *H*. *hainanensis* is most likely due to a severe demographic bottleneck, as this species has undergone approximately a 70% population reduction over the last three hundred years [16]. On Hainan Island, deforestation is greatly accelerated in the 20th century. About 80% to 95% of the primary forests were destroyed due to massive logging for timber, transitions to rubber tree and *Eucalyptus* plantations, as well as urban expansion [15, 53]. The *H*. *hainanensis* populations would shrink proportionally, perhaps even more, due to the high quality of its timber.

A population bottleneck was manifested by the qualitative graphical method based on the distorted distribution of allele frequencies in *H*. *hainanensis* populations (Fig 4). In non-bottlenecked populations near mutation-drift equilibrium, selectively neutral loci are expected to have a large proportion of alleles at low frequency. However, in recently bottlenecked populations, the expected distribution of allele frequencies would be distorted, with fewer alleles in the low frequency class (<0.1) than in one or more intermediate frequency classes [44, 54]. The graphical method makes no assumption about the ploidy of the studied species [44] and thus could be used in cases where common bottleneck testing tools, such as BOTTLENECK [55], could not be applied. We found a large number of low-frequency alleles (<0.1) are maintained in populations of the two non-threatened *Shorea* species. The ratio of the number of alleles at low frequency (<0.1) to the number of alleles at intermediate frequency (0.1–0.2) is 11.35 for *S*. *leprosula* and 7.10 for *S*. *macrophylla*. However, the ratios are 3.86 and 1.0 for *V*. *seychellarum* and *H*. *hainanensis*, respectively. Obviously, many low-frequency alleles have been lost during recent bottlenecks in the populations of *V*. *seychellarum* and *H*. *hainanensis*. A higher proportion of low-frequency alleles are maintained in *V*. *seychellarum* than in *H*. *hainanensis*, which may arise from different sample sizes used for the two species. Since the graphical method requires only samples of 5 to 20 polymorphic loci and approximately 30 individuals, the SSR data used in this study should be adequate [44]. To conclude, the *H*. *hainanensis* populations on Hainan Island may have experienced a recent demographic bottleneck, resulting in relative fewer alleles at low frequency and lower genetic diversity detected in this species. The genetic diversity of *H*. *hainanensis* in Northeast Vietnam was recently evaluated using 10 SSR markers [56]. The populations collected in Northeast Vietnam (*H*_e_ = 0.382, allelic richness = 2.08) and Hainan Island have similar levels of genetic variation (Table 3). A population bottleneck was reported and suggested to be responsible for the low genetic diversity of these populations [56].

When the size of a population is drastically reduced, genetic drift may override natural selection and act as the main evolutionary force, leading to a loss of genetic variation [3]. Moreover, inbreeding is inevitable in small populations [3, 5]. The inbreeding level of a population can be quantified with the inbreeding coefficient (*G*_is_). In this study, *G*_is_ ranged from 0.138 to 0.408, higher than the *G*_is_ estimates for many other dipterocarps [48–50]. The proposed bottleneck event in *H*. *hainanensis* and resulting population fragmentation likely produced small and isolated populations, in which enhanced inbreeding would be expected [3]. Significant deviation from HWE was detected in a large portion of the loci from the 10 study populations (Table 3). Nonrandom mating may be the main cause of the departure from HWE in those populations. In conclusion, the low genetic diversity, increased inbreeding and deviations from HWE found in the *H*. *hainanensis* populations on Hainan Island are typical of endangered species, and are likely the consequence of intensified inbreeding and genetic drift acting in small *H*. *hainanensis* populations.

### Population genetic structure and differentiation

Three genetic groups were identified in *H*. *hainanensis* using both Bayesian model-based clustering and principal coordinate analysis (PCoA) (Figs 2 and 3). The assignment of individuals to genetic groups was consistent between the two methods. Additionally, the topology of a neighbor-joining tree was concordant with the clustering results from STRUCTURE and the PCoA (S1 Fig). The three genetic clusters were widely distributed across the mountainous area of Hainan Island, without any clear geographical pattern (Fig 1). In addition, a Mantel test did not detect a correlation between genetic divergence (as Slatkin's linearized *F*_ST_) and geographical distance (S2 Fig), suggesting that genetic differentiation in *H*. *hainanensis* does not follow a pattern of isolation by distance. Such population genetic structure implies the existence of long-distance gene flow among populations before the species-wide bottleneck. Long-distance gene flow has been reported in *Neobalanocarpus heimii*, *Dipterocarpus tempheses* and several *Shorea* species [56, 57]. *Neobalanocarpus heimii* is an emergent tree species endemic to the Malay Peninsula. Using paternity analysis, the average distance of pollen flow in *N*. *heimii* was estimated to be 191 m, while several pollination events were found to exceed 400 m [58]. In addition, seeds produced by *N*. *heimii* may be transported by squirrels. Interestingly, as with *H*. *hainanensis*, no positive correlation between genetic relatedness and spatial distance was detected in *N*. *heimii*. Long-distance gene flow via pollen and seed migration are likely responsible for the weak genetic structure in this species [58]. In summary, the widespread geographical distribution of genetic subpopulations and the failure to find isolation by distance in *H*. *hainanensis* are likely due to long-distance gene flow among populations before the species-wide bottleneck.

Endangered dipterocarp species are generally highly differentiated across populations based on the analysis of SSR markers. For example, the overall *G*_ST_ across adult populations of the endangered *V*. *seychellarum* is 0.20 [23]. Habitat fragmentation and restricted gene flow were suggested as possible reasons for the strong genetic divergence of this species [23]. Genetic differentiation was quantified for a few *Hopea* species. A moderate level of differentiation between two geographically adjacent populations was revealed in *H*. *bilitonensis* (mean value of *G*_ST_ is 0.116), which is an extremely rare and predominantly selfing dipterocarp in Peninsular Malaysia [24]. Two threatened *Hopea* species were also reported to have low to moderate levels of genetic differentiation (*G*_ST_ = 0.009 for *H*. *chinensis*; *G*_ST_ = 0.102 for *H*. *odorata*) [56]. Compared to the three above *Hopea* species, *H*. *hainanensis* showed higher overall genetic divergence (*G*_ST_ = 0.229). The AMOVA analysis also revealed high differentiation among populations (*F*_ST_ = 0.23). Population genetic theory predicts that under Island model of complete isolation, reduction in population size could promote divergence among populations [59], which is likely the reason of elevated differentiation observed in *H*. *hainanensis*. However, other factors, such as the number of generations after population isolation (*t*), geographic distance between sampled populations, mating system and evolutionary history also influence the level of population divergence [46]. In summary, further research is needed to explore the effect of habitat fragmentation on genetic differentiation across *H*. *hainanensis* populations.

#### Implications for conservation

Because the loss of genetic variation is a major threat to the survival of endangered species, an important conservation action is to preserve and restore genetic variation [3, 60]. Three *H*. *hainanensis* populations, BW, FJ and DL, were found to have relatively lower levels of genetic variation. These three populations may therefore be more vulnerable to abiotic and biotic stresses and should be the focus of special conservation attention. Meanwhile, the BL, JF, JX, LM and QW populations had relatively higher levels of genetic diversity and contained more than one genetic subpopulation. These five populations could therefore serve as seed sources for the propagation of seedlings and saplings, to be used in the restoration of previously logged lowland rainforests on Hainan Island. The natural regeneration of *H*. *hainanensis* populations is challenging: seedlings and saplings grow very slowly and often fail to establish in the highly shaded understory. Therefore, selecting populations with high genetic diversity (e.g. BL, JF, LM and QW) to produce seedlings may facilitate the restoration of endangered *H*. *hainanensis* populations on Hainan Island.

## Supporting information

S1 File SSR genotypes of 76 *H*. *hainanensis* individuals used in this study. (XLSX)

S1 Table The BIC scores of the four polysomic inheritance models implemented in Polygene version 1.2. Two options, “Consider negative PCR” and “Consider selfing”, were combined along with optimal model selection.
(XLSX)

S2 Table Pairwise divergence among *Hopea hainanensis* populations based on Nei’s *G*_ST_. (XLSX)

S1 Fig Mantel test of the correlation between genetic distance (as Slatkin's linearized *F*_ST_) and the natural logarithm of geographical distance (km). (TIF)

S2 Fig Neighbor joining tree for individual samples based on Cavalli-Sforza’s (1967) genetic distance. Colored bars represent individual assignments by STRUCTURE when *K* = 3.
(TIF)
