# Supplementary figures and images for: Genetic diversity and population structure in the endangered tree Hopea hainanensis (Dipterocarpaceae) on Hainan Island, China
Source: PLoS One. 2020 Nov 30;15(11):e0241452. doi: 10.1371/journal.pone.0241452 (PMC7703895; doi:10.1371/journal.pone.0241452)

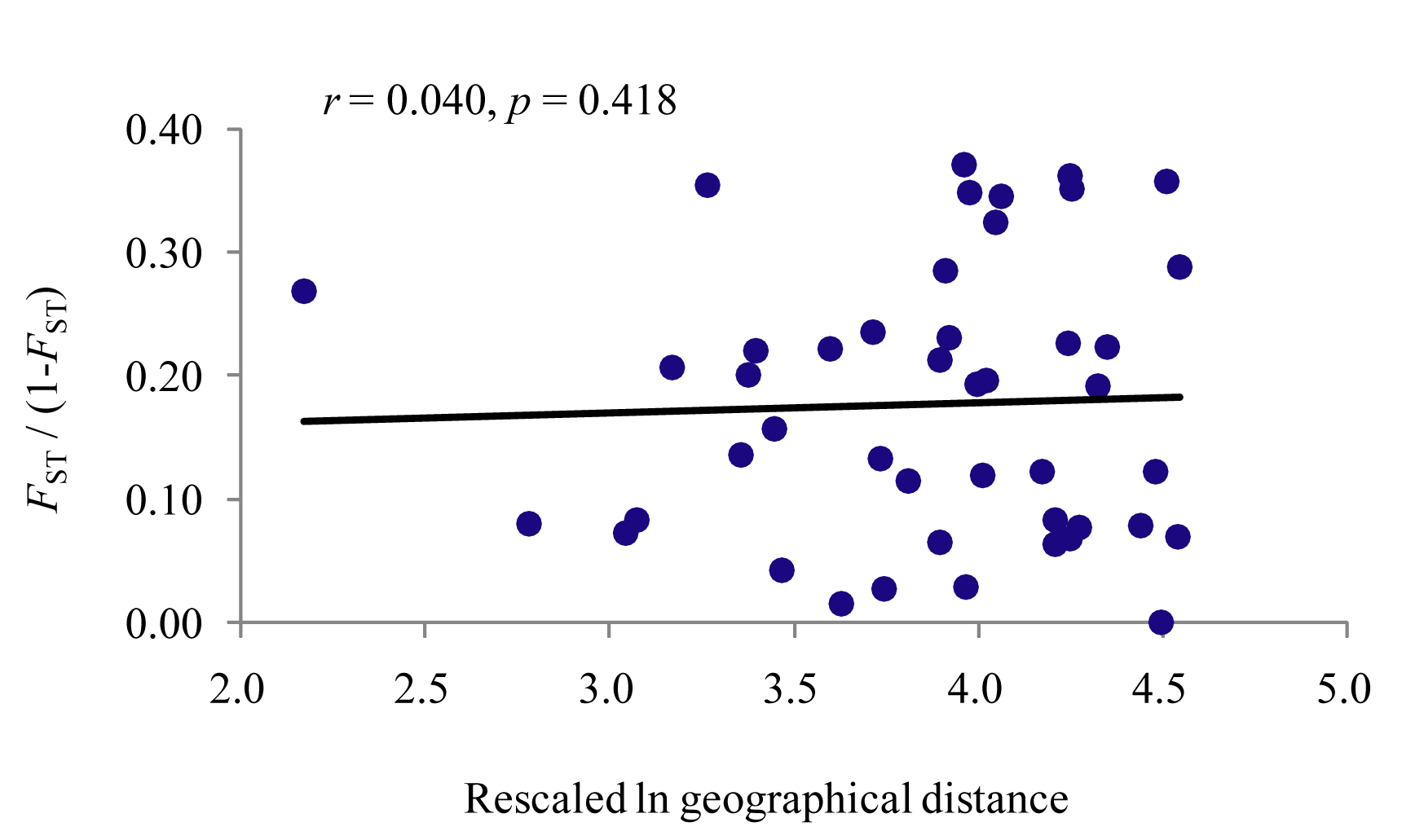

Supplement: S1 Fig — (TIF) [file pone.0241452.s004.tif]

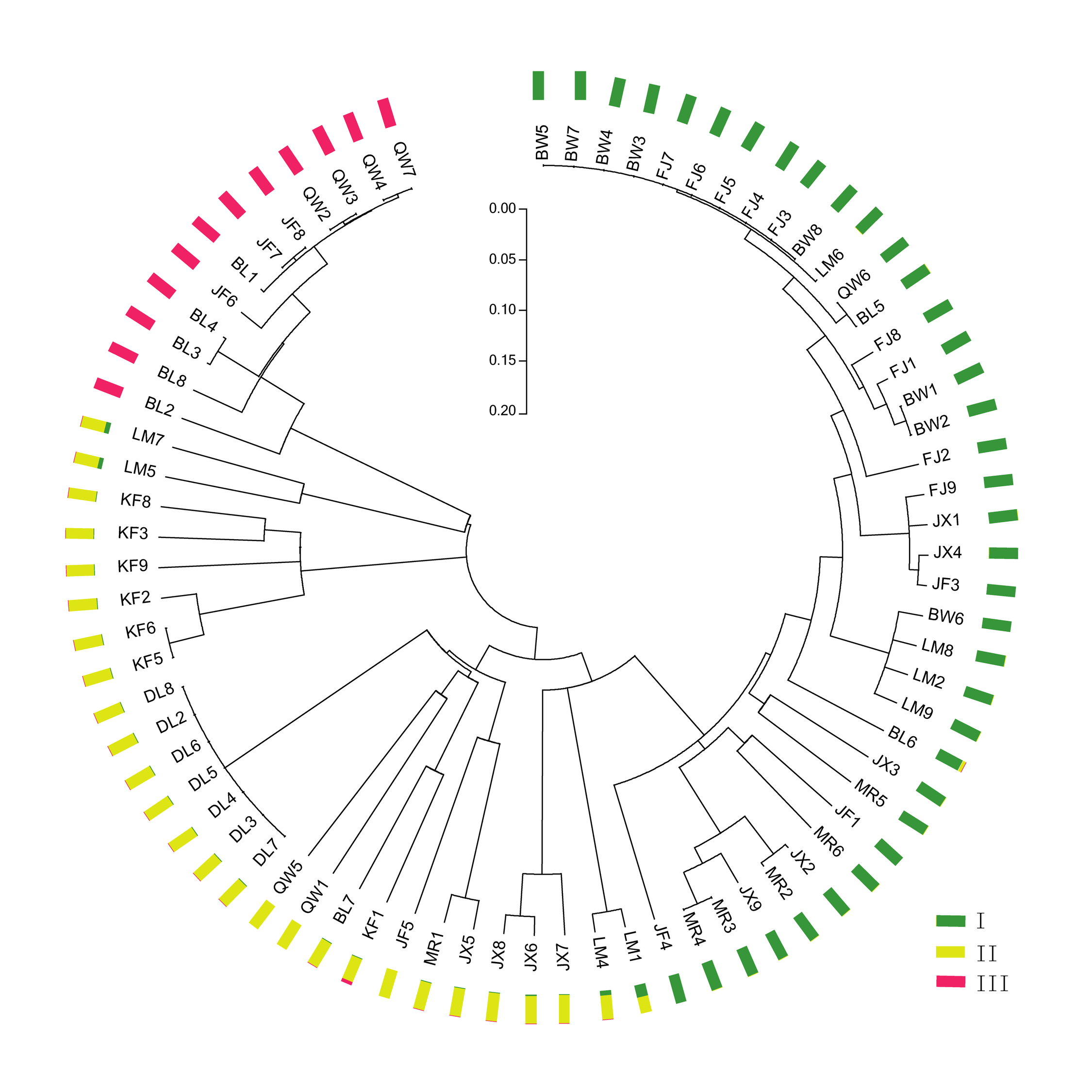

Supplement: S2 Fig — Colored bars represent individual assignments by STRUCTURE when K = 3. (TIF) [file pone.0241452.s005.tif]
